# Human resource implications of expanding latent tuberculosis patient care activities

**DOI:** 10.3389/fmed.2023.1265476

**Published:** 2024-01-12

**Authors:** Hannah Alsdurf, Andrea Benedetti, Tran Ngoc Buu, Menonli Adjobimey, Victoria J. Cook, Dina Fisher, Gregory Fox, Federica Fregonese, Panji Hadisoemarto, James Johnston, Richard Long, Joseph Obeng, Olivia Oxlade, Rovina Ruslami, Kevin Schwartzman, Erin Strumpf, Dick Menzies

**Affiliations:** ^1^Department of Epidemiology, Biostatistics and Occupational Health, McGill University, Montreal, QC, Canada; ^2^McGill International TB Centre, McGill University, Montreal, QC, Canada; ^3^Woolcock Institute of Medical Research, Hanoi, Vietnam; ^4^Programme National Contre la Tuberculose, Centre National Hospitalier Universitaire de Pneumo-Phtisiologie, Cotonou, Benin; ^5^Provincial Tuberculosis Services, British Columbia Centre for Disease Control, Vancouver, BC, Canada; ^6^Department of Medicine, University of British Columbia, Vancouver, BC, Canada; ^7^Division of Respiratory Medicine, University of Calgary, Calgary, AB, Canada; ^8^The University of Sydney Central Clinical School, The Faculty of Medicine and Health, The University of Sydney, Sydney, NSW, Australia; ^9^Department of Public Health, Faculty of Medicine, TB-HIV Research Center, Universitas Padjadjaran, Bandung, Indonesia; ^10^Department of Medicine, Faculty of Medicine and Dentistry, University of Alberta, Edmonton, AB, Canada; ^11^Chest Clinic, Komfo Anokye Teaching Hospital, Kumasi, Ghana; ^12^Respiratory Epidemiology and Clinical Research Unit (RECRU), McGill University, Montreal, QC, Canada; ^13^Department of Biomedical Sciences, Division of Pharmacology and Therapy, Faculty of Medicine, Universitas Padjadjaran, Bandung, Indonesia

**Keywords:** latent tubercolosis infection, time and motion studies, tuberculosis - epidemiology, health system strengthening, active tuberculosis (TB)

## Abstract

**Introduction:**

The World Health Organization (WHO) declared increasing services for latent tuberculosis infection (LTBI) a priority to eliminate tuberculosis (TB) by 2035. Yet, there is little information about thehuman resource needs required to implement LTBI treatment scale-up. Our study aimed to estimate the change in healthcare workers (HCW) time spent on different patient care activities, following an intervention to strengthen LTBI services.

**Methods:**

We conducted a time and motion (TAM) study, observing HCW throughout a typical workday before and after the intervention (Evaluation and Strengthening phases, respectively) at 24 health facilities in five countries. The precise time spent on pre-specified categories of work activities was recorded. Time spent on direct patient care was subcategorized as relating to one of three conditions: LTBI, active or suspected TB, and non-TB (i.e., patients with any other medical condition). A linear mixed model (LMM) was fit to estimate the change in HCW time following the intervention.

**Results:**

A total of 140 and 143 HCW participated in the TAMs during the Evaluation and Strengthening phases, respectively. Results from intervention facilities showed an increase of 9% (95% CI: 3%, 15%) in the proportion of HCW time spent on LTBI-related services, but with a corresponding change of -11% (95% CI: -21%, -1%) on active TB services. There was no change in the proportion of time spent on LTBI care in control facilities; this remained low in both phases of the study.

**Discussion:**

Our findings suggest that additional HCW personnel will be required for expansion of LTBI services to ensure that this expansion does not reduce the time available for care of active TB patients.

## Introduction

According to World Health Organization (WHO) estimates, there were over 10 million new cases of tuberculosis (TB) worldwide in 2021 (1, 2). It is further estimated that nearly 25% of the world’s population is latently infected with TB, or almost 2 billion people globally (1). In 2015, the WHO announced the End TB Strategy with the goal of ending TB by 2035 (i.e., an incidence of less than 10/100,000). The End TB strategy has three main pillars, one of which is to focus on integrated, patient-centered care and prevention (3). The WHO has further prioritized the identification and preventive treatment of people who are at high risk of latent TB infection (LTBI), of whom close household contacts (HHC) are the largest group (1). Following the United Nations High Level Meeting on TB in 2018, support was declared to increase the health workforce providing TB services as part of a larger commitment to strengthen public health systems (4).

While this focus on improving access to preventive TB services represent an important step towards TB elimination efforts, there is little published information on the workload for healthcare workers (HCW) currently providing TB care. Furthermore, staffing challenges in health facilities already exist, particularly in low- and middle-income countries (LMIC), that face a shortage of well-trained, qualified staff (5). Another key barrier to scale-up of health services globally is ineffective health service delivery, particularly in remote or rural areas where there are too few HCW for the demand on services (6, 7). In order to ensure high-quality, patient-centered care for scale-up of preventive services for LTBI, it is necessary to better understand how expanded services will affect human resource and staffing needs of healthcare facilities and providers.

A time and motion study (TAM) was performed to estimate the change in HCW time spent on patient care activities following a standardized intervention to improve the identification, diagnosis and treatment of household contacts with LTBI. Our study aimed to determine the change in proportion of HCW time devoted to three categories of patient care activities following the intervention: (1) LTBI; (2) active or suspected TB; and (3) non-TB.

## Materials and methods

### Parent study

Our study (the “TAM study”) was conducted as part of a pragmatic, cluster-randomized trial conducted in a total of 24 health facilities in 5 countries. Four health facilities were selected in a low TB incidence setting (Canada), and 20 health facilities were chosen in LMICs with intermediate to high TB incidence rates (2 sites in Benin, 2 in Ghana, 8 in Indonesia, and 8 in Vietnam) which is described in detail elsewhere (8–10). The overall objective of the parent trial was to strengthen the LTBI cascade of care for household contacts in these countries (9). At intervention sites, the study began with an Evaluation phase which included a standardized, retrospective review of patient registry data, from the identification and screening for LTBI through to starting and completing therapy. This cascade analysis, along with a standardized questionnaire, identified the steps in the LTBI cascade of care at each study site with the greatest losses of patients to address via interventions. During the Strengthening phase, intervention activities implemented included initial and in-service trainings for HCW on LTBI testing and treatment at all sites. Sites also identified local solutions to address cascade losses and improve the uptake of LTBI services (9). These included home visits to identify and test more household contacts, flipcharts for HCW education, SMS reminders for LTBI patients, or extended TB clinic hours to facilitate LTBI patient visits. The impact of the Strengthening phase activities on HCW time allocation was not hypothesized *a priori*, but was measured as part of this sub-study of the parent ACT4 trial. Control sites continued to provide TB services per standard programmatic care, based on national guidelines for testing (i.e., TST or IGRA) and treatment regimens, and did not receive the Evaluation nor the Strengthening activities given to intervention sites.

### Time and motion study

The TAM study used a cohort design with purposive sampling of different cadres of HCW providing TB care. Consenting HCW at all participating health facilities who worked at least one full day per week delivering TB care were eligible to participate in the TAM study. At each health facility, we aimed to include a minimum of ten HCW, and at least three HCW in each cadre: (1) doctors; (2) nurses; (3) other HCW involved in TB care (i.e., social workers, health assistants, pharmacists, and community health workers).

The TAM consisted of a research assistant observing each participating HCW continuously, and noting down minute-by-minute each activity that the HCW performed throughout the day. TAMs were scheduled in advance with each HCW for a workday in which the HCW did not have any planned or likely changes in their normal patient care activities or clinical schedule (such as leaving early to attend a personal appointment), and this was confirmed at the start of the TAM day. When the same HCW was not available for a TAM during the Strengthening phase, we attempted to replace that HCW with another HCW of the same cadre.

### Measurement instruments

The work tasks performed by HCW in the health facility were categorized into three main types of activities: (1) Direct patient care (i.e., any face-to-face encounter or phone call with a patient); (2) Other clinical activities (i.e., charting, dictations, reviewing laboratory results or radiographs); and (3) Training or administrative tasks (i.e., supervising trainees, meetings, or emails). Time spent on breaks (i.e., restroom, meals or personal phone calls) was recorded during the TAMs but was removed from the analyses. Time spent on direct patient care was sub-categorized based on how it related to patients with one of three conditions: (1) LTBI; (2) active or suspected TB; and (3) non-TB (i.e., patients with any other medical condition). When a visit included multiple patients at the same time (parent with active TB and a child contact) the total visit time was divided in half, with 50% of the total visit time attributed to active TB and 50% attributed to LTBI.

### Data collection

Data collection was conducted between January 2017 and December 2018. To ensure standardized measurements, all research staff performing the TAMs received initial, and refresher training from one investigator (HA) on how to observe and record HCW time using standard data collection forms and properly classify and code each observation. All data was recorded on paper data collection sheets and then, de-identified data was transferred to Excel spreadsheets with pre-specified drop-down menus. Verbal consent was obtained from all HCW to permit research staff to observe their daily work activities. Research staff conducting the TAMs did not enter patient rooms during encounters with observed workers.

### Breakdown of time spent by personnel

Time spent on each of the three categories of activities (direct patient care, other clinical activity, and training/administrative tasks) was calculated as a proportion of total time worked on the day of observation (TAM day) for each participating HCW [Equation #1: *Proportion of direct patient care on LTBI = Time on LTBI/total time on direct patient care (Active TB + LTBI + Non-TB)*]. We also calculated the proportion of time providing care for patients divided into three categories: active TB, LTBI, or non-TB, as a proportion of total time spent providing direct patient care. The time spent on other clinical activities was apportioned to the three types of patients based on the proportion of direct patient care time. Total patient care time for the three categories of types of patients was calculated as observed time on direct patient care plus the apportioned time on other clinical activities (Equation #2: *Total LTBI patient care time = Total hours on LTBI +[(Proportion of time on LTBI (equation 1 above)) × (total time on other clinical activities)]*). Finally, the total time for each type of patient (i.e., active TB, LTBI, and non-TB) was divided by total patient care time (i.e., direct patient care plus other clinical activity) to calculate the proportion of total patient care time for each type of patient (Equation #3: *Proportion of total LTBI patient care time = Total LTBI patient care time (equation 2)/[total time on direct patient care + total time on other clinical activities (all types of patients)]*).

### Analyses

#### Descriptive statistics

Characteristics of all HCW who participated in TAMs in the Evaluation phase were compared to HCWs who particiapated in the Strengthening phase. Boxplots were used to describe changes between Evaluation and Strengthening phases in the proportion of HCWs’ time spent on the three categories of work activities, as well as the three categories of patient type. These were shown separately for intervention and control sites. The mean and median number of hours worked, stratified by intervention and control sites, was estimated for the following categories: (1) Total time worked during the TAM day; and time spent on: (2) direct patient care; (3) other clinical activities; and (4) Training/administrative tasks.

#### Statistical analysis

A linear mixed model (LMM), by site and study phase, was fit for all categories of HCW time allocation including: (1) Total time worked; (2) direct patient care; (3) other clinical activities; (4) training/administrative tasks; (5) LTBI patient care; (6) active TB patient care; and (7) non-TB patient care. For each model, the dependent variable was the number of hours worked in the given category, and the model included terms for phase, intervention and their interaction. A random intercept for site was included to account for correlation between healthcare workers in the same facility, and a random intercept for healthcare worker was included to account for correlation between observations on the same worker.

Linear mixed models were also fit for each type of patient (i.e., active TB, LTBI, and non-TB) for intervention and control sites by study phase for proportion of total patient care time (i.e., direct patient care and other clinical activities). As above, for each model, the dependent variable was the proportion of hours worked in the given category, and the model included terms for phase, intervention and their interaction. All models included a random intercept for site to account for correlation between healthcare workers in the same facility, and a random intercept for healthcare worker to account for correlation between the observations of the same worker. From these models, the difference in proportion of time before vs. after the intervention was estimated for control and intervention arms separately. The effect of the intervention was estimated using a model of the difference in the changes in the proportion of healthcare worker time between the intervention and control groups.

### Sensitivity analyses

To detect the role of subgroup characteristics, sensitivity analyses were done adjusting for the following covariates: (1) sex, (2) TB-specific job position, (3) HCW cadre (i.e., doctor, nurse, other HCW), (4) country, (5) type of setting based on country level income and TB-incidence [i.e., high-TB incidence and low-middle income (Benin, Ghana, Indonesia, and Vietnam) vs. low-TB incidence and high-income (Canada)]. Interactions, defined *a priori*, were considered between type of setting and HCW sex, cadre and TB-specific job. Data were analyzed using SAS version 9.4 (SAS Institute, Cary, United States).

Additional sensitivity analyses were performed for the subset of HCW who participated in TAMs in both the Evaluation and Strengthening phases (i.e., within-subject analysis). In this analysis, we calculated the change in proportion of time for each HCW, then calculated the mean difference across all HCW. Linear mixed models for the change in proportion of total patient care time (by type of patient) were run by site and study phase, then the differences between intervention and control arms were calculated in the same manner as for the full dataset.

### Ethics

The Ethics Review Board of the Research Institute of the McGill University Health Centre, and the Research ethics boards at all participating sites approved this study.

## Results

**Figure 1:**
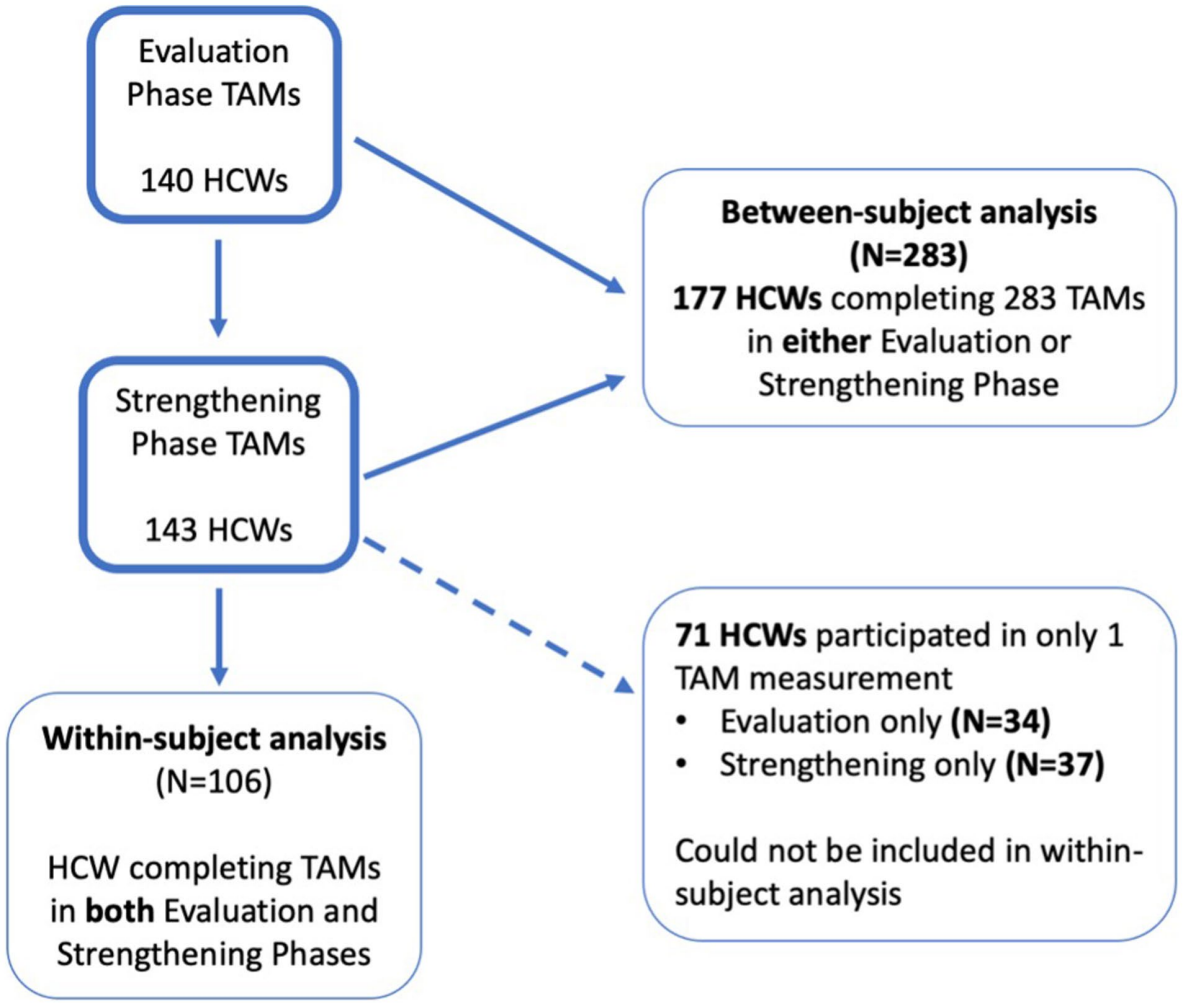
Flow diagram of HCW participating in time and motion study (TAMs).

In total, 140 and 143 HCW participated in the TAMs in the Evaluation and Strengthening phases, respectively (main analysis). HCW who participated in the Evaluation phase were largely similar to those who participated in the Strengthening phase (Table 1). Of these, 106 HCW completed TAMs in both study phases and were included in the sensitivity analyses (Figure 1; Table 2). There were more doctors and other HCW who had TAMs in both study phases compared to those who only participated in TAMs in one phase (Table 2). Indonesia had significantly more HCW who participated in TAMs during only one study phase, compared to HCW who had TAMs in both phases (Table 2).

**Table 1:** Characteristics of HCWs^1^ participating in the time and motion study (TAMs): comparison of all HCWs participating in either Evaluation or Strengthening phases.

	Evaluation phase (*N* = 140)	Strengthening phase (*N* = 143)
*Sex*		
Male	45 (32%)	42 (29%)
Female	95 (68%)	101 (71%)
*TB specific role*		
Yes	87 (62%)	89 (62%)
No	53 (38%)	54 (38%)
*HCW category*		
Doctor	73 (52%)	70 (49%)
Nurse	56 (40%)	63 (44%)
Other HCW	11 (8%)	10 (7%)
*Type of site*		
Intervention	63 (45%)	66 (46%)
Control	77 (55%)	77 (54%)
*Country*		
Benin	18 (13%)	18 (12%)
Canada	39 (28%)	41 (29%)
Ghana	14 (10%)	13 (9%)
Indonesia	28 (20%)	30 (21%)
Vietnam	41 (29%)	41 (29%)

^1^Data presented for all HCWs who participated in TAMs during that phase.

**Table 2:** Descriptive characteristics of HCWs participating in TAMs in both Evaluation and Strengthening phases to HCWs with TAMs only in one phase.

	Within-subject^1^ analysis (BOTH Evaluation and Strengthening) (*N* = 106)	HCW with only 1 TAM (Evaluation OR Strengthening) (*N* = 71)
*Sex*		
Male	36 (34%)	18 (25%)
Female	70 (66%)	53 (75%)
*TB specific role*		
Yes	66 (62%)	45 (63%)
No	40 (38%)	26 (37%)
*HCW category*		
Doctor	58 (55%)	30 (42%)
Nurse	39 (37%)	39 (55%)
Other HCW	9 (8%)	2 (3%)
*Type of site*		
Intervention	49 (46%)	32 (45%)
Control	57 (54%)	39 (55%)
*Country*		
Benin	15 (15%)	6 (8%)
Canada	33 (31%)	14 (20%)
Ghana	10 (9%)	7 (10%)
Indonesia	11 (10%)	36 (51%)
Vietnam	37 (35%)	8 (11%)

^1^Within-subject – data presented for HCWs who participated in TAMs during both Evaluation and Strengthening phases.

**Figure 2:**
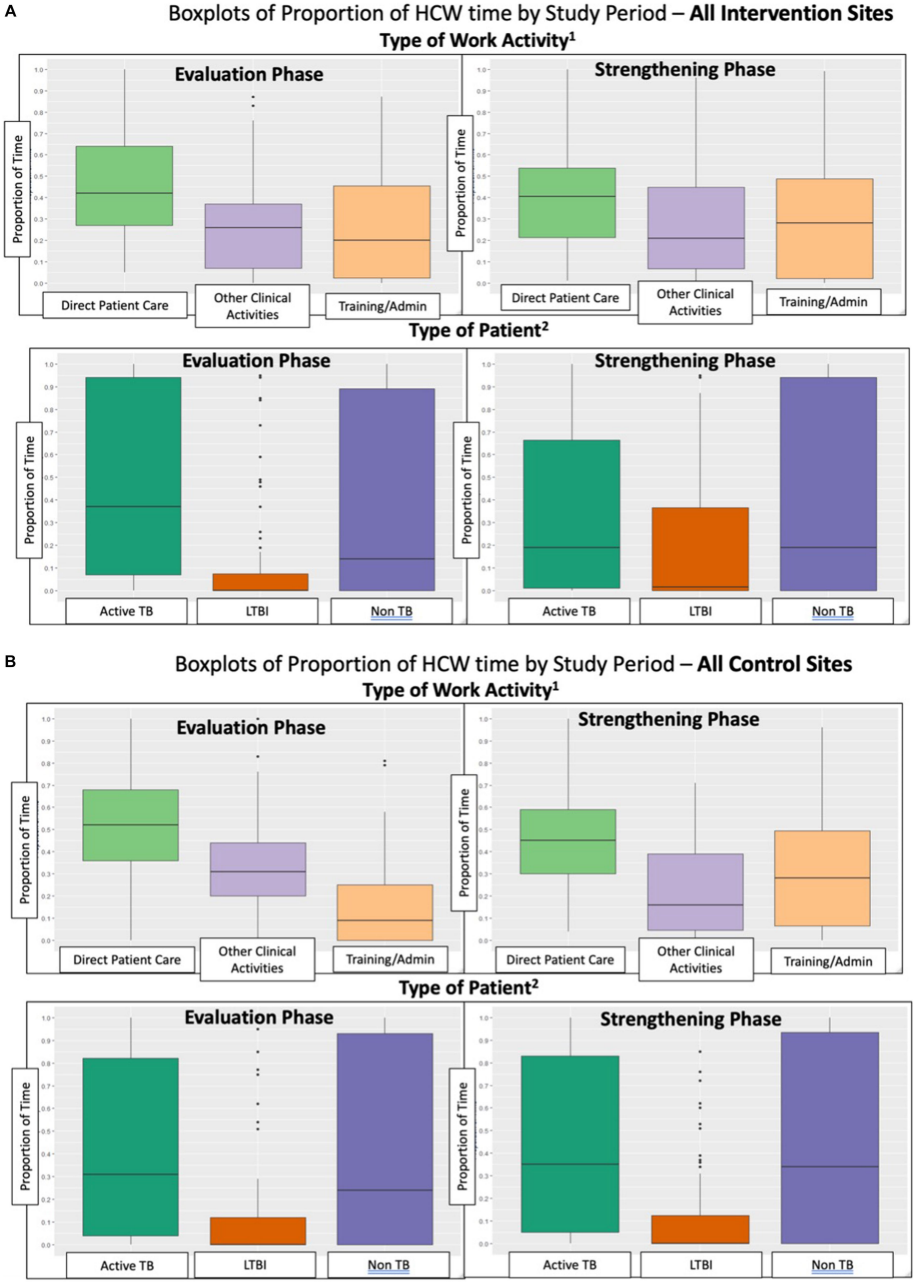
**(A)** Boxplots of proportion of HCW time by study period – all intervention sites. **(B)** Boxplots of Proportion of HCW time by study period – all control sites.

Overall, HCW worked approximately the same number of total hours per day in the Evaluation and Strengthening phases and there was not a significant difference in the change in total hours worked between control and intervention sites (Table 3). HCW time spent on direct patient care decreased from the Evaluation to Strengthening phase, but there was no significant difference in this change between control and intervention sites (Table 3; Figure 2). Time on training and administrative tasks increased in control and intervention sites, with control sites increasing training/administrative time significantly more than intervention sites (Table 3; Figure 2).

**Table 3:** Average change in the time (hours) worked on TAM study day for all HCWs participating in TAMs^1^ - by type of work activity^2^.

	Control arm		Intervention arm	
	Evaluation phase	Strengthening phase	Evaluation phase	Strengthening phase
*Total time (hours)*				
Total HCW time worked on TAM day	5.28 (4.60, 5.96)	4.77 (4.09, 5.44)	5.42 (4.72, 6.12)	5.19 (4.49, 5.89)
Within-site change between Evaluation and Strengthening phases	−0.51 (−1.00, −0.03)^*^		−0.23 (−0.77, 0.30)	
Between-site difference in change (hours)	0.28 (−0.44, 1.01)			
*Direct patient care (hours)*				
HCW time (hours) on direct patient care	2.91 (2.49, 3.33)	2.09 (1.67, 2.50)	2.76 (2.32, 3.20)	2.27 (1.83, 2.70)
Within-site change between Evaluation and Strengthening phases	−0.82 (−1.18, −0.46)^**^		−0.49 (−0.89, −0.09)^*^	
Between-site difference in change (hours)	0.33 (−0.21, 0.87)			
*Other clinical activities (hours)*				
HCW time (hours) on other clinical activities	1.95 (1.46, 2.43)	1.17 (0.69, 1.65)	1.44 (0.94, 1.95)	1.17 (0.67, 1.68)
Within-site change between Evaluation and Strengthening phases	−0.78 (−1.15, −0.41)^**^		−0.27 (−0.67, 0.14)	
Between-site difference in change (hours)	0.51 (−0.04, 1.06)			
*Training/administrative tasks (hours)*				
HCW time (hours) on training/administrative tasks	0.49 (0.02, 0.95)	1.55 (1.09, 2.02)	1.25 (0.76, 1.73)	1.75 (1.27, 2.23)
Within-site change between Evaluation and Strengthening phases	1.06 (0.69, 1.44)^**^		0.50 (0.09, 0.91)^*^	
Between-site difference in change (hours)	−0.56 (−1.12, −0.01)^*^			
*Patient care in latent tuberculosis infection (LTBI) (hours)*				
HCW time (hours) on LTBI patient care	0.59 (0.05, 1.14)	0.30 (−0.24, 0.85)	0.64 (0.07, 1.20)	0.80 (0.24, 1.37)
Within-site change between Evaluation and Strengthening phases	−0.29 (−0.62, 0.04)		0.16 (−0.20, 0.52)	
Between-site difference in change (hours)	0.45 (−0.03, 0.95)			
*Patient care in active TB (hours)*				
HCW time (hours) on active TB patient care	2.16 (1.54, 2.77)	1.36 (0.75, 1.97)	2.10 (1.46, 2.75)	1.54 (0.90, 2.19)
Within-site change between Evaluation and Strengthening phases	−0.80 (−1.24, −0.35)^**^		−0.56 (−1.05, −0.08)^*^	
Between-site difference in change (hours)	0.24 (−0.42, 0.89)			
*Patient care in non-TB (hours)*				
HCW time (hours) on non-TB patient care	2.02 (1.52, 2.52)	1.47 (0.97, 1.97)	1.43 (0.90, 1.96)	1.10 (0.58, 1.63)
Within-site change between Evaluation and Strengthening phases	−0.55 (−0.94, −0.16)^**^		−0.33 (−0.75, 0.10)	
Between-site difference in change (hours)	0.22 (−0.35, 0.80)			

^1^Proportion of total patient care time which includes direct patient care and other clinical activities. ^2^Data presented for all HCWs who participated in TAMs in either Evaluation or Strengthening phase. *Statistically significant difference at *p* < 0.05; **Statistically significant difference at *p* < 0.01.

At intervention sites, there was a significant increase of 9% (95% CI: 3, 15%) in the proportion of total patient care time spent on LTBI patient care, and a significant change of −11% (95% CI: −21, −1%) in the proportion of HCW patient care time spent on active TB patients (Table 4). The baseline proportion of total patient care time spent on LTBI activities averaged 40% at intervention sites in Canada vs. an average of 1% in high-TB burden settings (LMICs) (data not shown in tables). The proportion of patient care time spent on LTBI activities, relative to baseline, increased by 10% in the Strengthening phase in all intervention sites in all countries. There was a significant difference between control and intervention sites in the change from Evaluation to Strengthening phases for LTBI-related activities of 11% (95% CI: 3, 19%), and a similar magnitude although non-significant change in proportion of time for active TB care of −12% (95% CI: −26, 1%). As seen in Table 4 and Figure 2, the proportion of total patient care time spent on patients with other (non-TB) health problems did not change significantly. Sensitivity analyses was performed among the 106 HCW who participated in the TAMs in both Evaluation and Strengthening phases (i.e., two TAMs per HCW), and showed similar results (Table 5).

**Table 4:** Average change in proportion of total patient care time^1^ for all HCW participating in TAMs^2,3^ – by type of patient.

	Control arm		Intervention arm	
	Evaluation phase	Strengthening phase	Evaluation phase	Strengthening phase
*Latent TB infection (LTBI)*				
Proportion of HCW time on LTBI patient care	0.09 (0.01, 0.18)	0.07 (−0.01, 0.16)	0.08 (−0.01, 0.17)	0.17 (0.08, 0.26)
Within-site change between Evaluation and Strengthening phases	−0.02 (−0.07, 0.03)		0.09 (0.03, 0.15)^**^	
Between-site difference in change	0.11 (0.03, 0.19)^**^			
*Active TB*				
Proportion of HCW time on active TB patient care	0.40 (0.27, 0.53)	0.41 (0.29, 0.54)	0.49 (0.37, 0.62)	0.38 (0.26, 0.51)
Within-site change between Evaluation and Strengthening phases	0.01 (−0.08, 0.10)		−0.11 (−0.21, −0.01)^*^	
Between-site difference in change	−0.12 (−0.26, 0.01)			
*Non-TB*				
Proportion of HCW time on Non-TB patient care	0.50 (0.36, 0.65)	0.48 (0.34, 0.63)	0.44 (0.29, 0.59)	0.46 (0.31, 0.61)
Within-site change between Evaluation and Strengthening phases	−0.02 (−0.10, 0.07)		0.02 (−0.07, 0.11)	
Between-site difference in change	0.04 (−0.08, 0.16)			

^1^Proportion of total patient care time which includes direct patient care and other clinical activities. ^2^Data presented for all HCWs who participated in TAMs in either Evaluation or Strengthening phase. ^3^HCWs time was estimated via linear mixed models (LMM) that accounted for clustering. ^*^Statistically significant difference at *p* < 0.05; ^**^Statistically significant difference at *p* < 0.01.

**Table 5:** Average change in proportion of total patient care time^1^ for HCW participating in TAMs in both Evaluation and Strengthening phases^2,3^ – by type of patient.

	Control arm		Intervention arm	
	Evaluation phase	Strengthening phase	Evaluation phase	Strengthening phase
**Within-subject** ^**2**^ **analysis**				
*Latent TB infection (LTBI)*				
Proportion of HCW time on LTBI patient care	0.12 (0.02, 0.23)	0.09 (−0.01, 0.20)	0.11 (0.01, 0.21)	0.21 (0.11, 0.31)
Within-site change between Evaluation and Strengthening phases	−0.03 (−0.09, 0.03)		0.10 (0.03, 0.16)^**^	
Between-site difference in change	0.13 (0.04, 0.22)^**^			
*Active TB*				
Proportion of HCW time on active TB patient care	0.42 (0.27, 0.57)	0.44 (0.29, 0.59)	0.55 (0.40, 0.70)	0.43 (0.29, 0.58)
Within-site change between Evaluation and Strengthening phases	0.02 (−0.08, 0.12)		−0.12 (−0.22, −0.01)^*^	
Between-site difference in change	−0.14 (−0.28, 0.01)			
*Non-TB*				
Proportion of HCW time on non-TB patient care	0.52 (0.37, 0.66)	0.49 (0.34, 0.63)	0.42 (0.27, 0.57)	0.42 (0.27, 0.57)
Within-site change between Evaluation and Strengthening phases	−0.03 (−0.11, 0.05)		0.00 (−0.08, 0.09)	
Between-site difference in change	0.03 (−0.08, 0.16)			

^1^Proportion of total patient care time which includes direct patient care and other clinical activities. ^2^Within-subject – Data presented for HCWs who participated in TAMs during both Evaluation and Strengthening phases. ^3^HCWs time was estimated via linear mixed models (LMM) that accounted for clustering. ^*^Statistically significant difference at *p* < 0.05. ^**^Statistically significant difference at *p* < 0.01.

## Discussion

Results from our study demonstrated that an intervention to improve LTBI services resulted in a 9% (or 11%) increase in the proportion of HCW time providing LTBI-related patient care; this corresponds to approximately one additional hour of work per day. No change in HCW time allocation was shown at control sites. Since additional staff were not hired to perform these LTBI-related tasks, the additional time for the LTBI-related patient care activities was associated with a reduction in time spent on active TB patient care, an unintended and potentially important negative impact of the intervention.

### Limitations

TAMs are designed to capture repetitive work tasks (11) but HCW often have substantial day-to-day variability in the work tasks they perform. Since TAMs require an external observer for an entire workday, they are costly to perform and so it was not feasible for this study to include TAMs on each HCW more than once in the two phases of the parent trial. Hence, we did not capture the potential day-to-day variability in the hours and type of work and may have missed time spent on LTBI or active TB on other workdays. As well, HCW time on administrative tasks such as meetings, clerical work or end of year reporting increased significantly at control sites. We could find no reason for this finding which highlights the limitations of the TAM methodology - that it can be difficult to select a day for observation that is truly typical of HCW time allocation over the long duration of this study. However, the large number of participating HCW in our sample should have reduced the likelihood of a systematic bias in any particular direction. Although we pre-selected the days in which HCW were most likely to perform TB-related work tasks, the amount of HCW time spent on active TB or LTBI patient care was completely dependent on work tasks required on the specific TAM day.

The Hawthorne effect may have influenced results, since HCW were directly observed and thus may have taken fewer breaks or spent more time on each patient encounter. In order to reduce any such potential effects, and to alleviate any concerns, HCW were informed that their supervisors would not have access to the TAM data and the observations would have no possible impact on their work performance evaluations. As well, the break time was removed from all analyses. Any effect of spending more time on each patient encounter should have been non-differential, as it would be expected to affect encounters with patients with all types of health problems, not just LTBI-related patient encounters.

### Strengths

A key strength of our study was that we recruited a total of 177 participants in Canada and LMIC. In 13 previous continuous TAM studies we identified (12–14), 10 followed fewer than 35 HCW, although one prior study performed TAMs with 104 HCW (15). These findings were similar to a systematic review of 11 continuous TAMs studies performed in the hospital setting in which no more than 35 HCW were observed (16). Our study recruited many HCW to participate in multiple TAM measurements; over half of the HCW (60%) had TAMs in both the Evaluation and Strengthening phases. At participating African sites (Benin and Ghana) our TAM study had impressive recruitment, with >80% of TB-clinic HCW staff members participating, providing good representativeness. Most TAM studies do not follow HCW prospectively (12–16), thus another strength of our study was that we were able to capture average changes in proportion of HCW time following the intervention by collecting TAMs prospectively at two time points.

By conducting TAMs in the Evaluation and Strengthening phases, we were able to quantify changes in HCW proportion of time for each type of work activity, and patient type. This allowed us to estimate the increase in proportion of HCW time on LTBI service provision, as well as the negative impacts on care for patients with other health problems, as a result of the intervention. Results from the linear mixed models, accounting for clustering at the site level, showed a significant increase in the proportion of total patient care time on LTBI-related patient care, as well as a corresponding decrease in the proportion of total patient care time on active TB patient care. Although baseline proportion of total patient time on LTBI services was quite different between low- and high-TB burden settings, a consistent increase of ~10% of time was shown in all sites following strengthening activities. This finding indicates that locally driven solutions to improve LTBI services were successful at improving LTBI care. However, it is key to note that since these were solutions chosen and directed by local teams, there is limited generalizability to other settings, even within the same country. This finding highlights the need for additional studies across many settings to better understand context-specific drivers of uptake for healthcare interventions and services (17). Lastly, the findings of the sensitivity analyses conducted in those HCW with repeated TAM measurements (i.e., within-subject analysis) were consistent with the primary analyses that included all HCW highlighting the robustness of our results.

### Implications

Scale-up of LTBI services added almost an hour of LTBI-related work tasks each day to the HCW observed in our study on TAM days. This increase in HCW time corresponded to a significant increase of 60 household contacts per 100 active TB patients who initiated preventive treatment for LTBI following the intervention in the parent trial (10). Yet since no personnel were added, and HCW typically worked the same number of hours per day, this increased time for LTBI patient care was inevitably at the cost of reduced time for other patient care; notably care for patients with active TB. Shortages of HCW staffing for TB programs has long been a key challenge in many LMICs, but this was exacerbated by the COVID pandemic. Despite the decrease in HCW time spent on patients with active TB, this was not due to fewer persons with active TB since the parent trial showed that, the number of persons diagnosed with active TB remained the same following the intervention (9). Hence HCWs simply spent less time per person with active TB in order to prioritize LTBI patient care resulting from the objective of improving LTBI services at intervention sites.

The WHO target to expand LTBI services globally can be expected to have a major impact on the workload of HCW providing TB care. A recent review of the evidence of the impact of the COVID-19 pandemic on TB outcomes highlighted that across a number of high-TB burden countries showed 30–70% relative declines in preventive therapy enrolment (18). Additionally, many budgets for TB funding and healthcare personnel have been reduced or diverted to other services due to the pandemic (18, 19). Globally there have been significant decreases in the number of reported TB cases, as well as preventive TB services offered, due to these diversions of healthcare resources (20). The COVID-19 pandemic is one clear example of the impacts of task-shifting, moving limited personnel from one priority area of healthcare services to another. However, task-shifting can result from the addition of any new programme without increasing the available human resources or HCW. Our results suggest that additional human resources would be needed to provide LTBI services even with pre-pandemic TB clinic staffing levels, particularly for health facilities in LMIC with a high-TB burden. The estimated HCW needs could be based on the numbers of active TB patients and household contacts who will require screening and potentially treatment. But given the cuts to funding in many high-TB burden settings, which often already faced HCW shortages and TB-dedicated staff, the human resource needs will not be adequate to ensure quality care for both LTBI and active TB patients.

Lessons learned from the COVID-19 pandemic have included opportunities for synergies across disease silos for more holistic healthcare provision. Namely, shifts in the availability of virtual or telehealth visits, and the use of digital screening or contact tracing apps for COVID-19 patients are exciting areas for expansion to TB patient care and preventive treatment delivery options (19). These options have the added benefit of improving the quality and ease of care for patients and their families, while potentially decreasing the burden on HCW. However, additional, on-going work is needed to quantify and better understand where these solutions and technologies can be leveraged and what the actual impacts are on HCW time and effort for patient care.

## Conclusion

Both the United Nations’ declaration on TB and the WHO’s End TB Strategy have called for major expansions to LTBI services in TB programs globally (3, 4). The majority of efforts will need to be directed to identifying, testing and treating the estimated 20 million household contacts of people with active TB (4). Scale-up of LTBI services will require well-staffed national TB programs to conduct all the required work activities. TB programs globally need to assess the human resources requirements for expanded LTBI services to ensure scale-up does not come at the expense of quality care, particularly for active TB patients. Accurate estimation of the human resource needs to perform this additional work load, particularly given local staffing changes resulting from the pandemic, will be key to national TB programs’ ability to provide the increased LTBI services. This study contributes estimates of the HCW time allocation and workload needs to provide this patient care.

## Data availability statement

The raw data supporting the conclusions of this article will be made available by the authors, without undue reservation.

## Ethics statement

The studies involving humans were approved by Ethics Review Board of the Research Institute of the McGill University Health Centre. The studies were conducted in accordance with the local legislation and institutional requirements. The participants provided their written informed consent to participate in this study.

## Author contributions

HA: Conceptualization, Data curation, Formal analysis, Investigation, Methodology, Project administration, Writing – original draft, Writing – review & editing. AB: Formal analysis, Methodology, Software, Writing – review & editing. TB: Data curation, Investigation, Project administration, Writing – review & editing. MA: Data curation, Project administration, Writing – review & editing. VC: Project administration, Writing – review & editing. DF: Project administration, Writing – review & editing. GF: Investigation, Methodology, Project administration, Writing – review & editing. FF: Formal analysis, Methodology, Project administration, Writing – review & editing. PH: Investigation, Methodology, Project administration, Writing – review & editing. JJ: Project administration, Writing – review & editing. RL: Investigation, Supervision, Project administration, Writing – review & editing. JO: Data curation, Investigation, Project administration, Writing – review & editing. OO: Investigation, Methodology, Project administration, Writing – review & editing. RR: Project administration, Writing – review & editing. KS: Methodology, Writing – review & editing. ES: Methodology, Writing – review & editing. DM: Funding acquisition, Methodology, Project administration, Supervision, Writing – review & editing.
